# Prognostic Performance of Different Lymph Node Staging Systems in Patients With Small Bowel Neuroendocrine Tumors

**DOI:** 10.3389/fendo.2020.00402

**Published:** 2020-07-07

**Authors:** Sujing Jiang, Lihao Zhao, Congying Xie, Huafang Su, Ye Yan

**Affiliations:** ^1^Department of Radiation and Medical Oncology, The First Affiliated Hospital of Wenzhou Medical University, Wenzhou, China; ^2^Department of Gastroenterology, The First Affiliated Hospital of Wenzhou Medical University, Wenzhou, China

**Keywords:** small bowel neuroendocrine tumor, the log odds of positive lymph nodes, lymph node ratio, survival analysis, prognosis

## Abstract

**Background:** The prognostic significance of the lymph node (LN) classification for small bowel neuroendocrine tumors (SBNETs) remains unknown. The aim of the present study was to evaluate and compare the prognostic assessment of different LN staging systems.

**Methods:** Patients with SBNETs were identified from the Surveillance, Epidemiology, and End Results (SEER) database. The X-tile program was used to determine the cutoff value of the resected lymph nodes (RLNs), negative lymph nodes (NLNs), lymph node ratio (LNR), and the log odds of positive lymph nodes (LODDS). Survival analyses were performed using Kaplan–Meier curves with log-rank test. Logistic regression analysis was used to evaluate the differences between different periods. Univariate and multivariate Cox proportional hazards models were used to assess the prognostic value of different LN staging systems on cause-specific survival (CSS). The relative discriminative abilities of the different LN staging systems were assessed using the Akaike information criterion (AIC) and the Harrell consistency index (HCI).

**Result:** A total of 3,680 patients were diagnosed with SBNETs between 1988 and 2014 from the SEER database. A significant difference over time (1988–1999 vs. 2000–2014) was seen in age (*P* <0.001), tumor differentiation (*P* <0.001), T stage (*P* <0.001), and RLN (*P* <0.001) subgroups. Multivariate Cox survival analysis identified that LN status stratified by the number of RLNs, NLNs, LNR, and LODDS all predicted CSS in patients with SBNETs (all *P* <0.05), whereas the number of positive lymph nodes (PLNs) failed (*P* = 0.452). When assessed using categorical variables, LODDS staging systems showed the best prognostic performance (HCI: 0.766, AIC: 7,575.154) in the whole population. Further analysis based on different RLNs after eliminating the missing data showed that when the RLNs are <12, the LODDS (HCI: 0.769, AIC: 1,088.731) maintained the best prognostic performance as well when the RLNs are ≥12 (HCI: 0.835, AIC: 825.692). Among patients with LNR scores of 0 or 1, there was a residual heterogeneity of outcomes that were better stratified and characterized by the LODDS.

**Conclusion:** LODDS was a better predicator of survival when LN status was stratified as a categorical variable and should be considered when assessing the prognosis of patients with SBNETs to allow a more reliable means to stratify patient survival.

## Introduction

Small bowel neuroendocrine tumors (SBNETs) comprise the third largest subgroup of gastrointestinal neuroendocrine tumors (NETs) (1). The incidence of SBNETs has tripled in the past three decades because of the development of radiological endoscopic procedures (2). Historically, the exact staging of SBNETs has been problematic for the characteristically indolent nature (3). The prognosis may vary from slowly to highly aggressive disease with remarkably different outcomes (4). This variability causes significant challenges in medical decision making and treatment. The lymph node (LN) involvement may have a substantial prognostic effect and important therapeutic implications in SBNETs (5). In the staging classification of the European Neuroendocrine Tumor Society (ENETS), the LN staging of SBNETs is classified as N0 or N1 disease depending on the absence or presence of LN involvement (6). The recently updated American Joint Committee on Cancer (AJCC) eighth edition classification stratified the previous N1 disease into N1 [number of positive lymph nodes (PLNs) <12) and N2 (PLNs ≥ 12 or large mesenteric mass) (7). However, the prognostic significance of these categories is not yet established. Therefore, increasing attention has been paid to LN status, which can be evaluated by the number of resected lymph nodes (RLNs), PLNs, negative lymph nodes (NLNs), lymph node ratio (LNR), and the log odds of positive lymph nodes (LODDS). Compared to a single parameter, LNR showed its advantage in pancreatic (8), colon (9), breast (10, 11), and ovarian cancers (12). Another parameter, LODDS, is defined as the log[(PLN + 0.5)/(NLN + 0.5)] (13, 14). A value of 0.5 is added to both numerator and denomination to avoid singularity (15, 16). The LODDS also showed superiority in predicting outcomes in bladder (17), colorectal (18), lung (19), ovarian (20), and esophageal (21) cancers. In this study, we performed a large population-based database investigation of the prognostic values of RLNs, PLNs, NLNs, LNR, and LODDS in SBNETs, which may inform treatment options and prognosis discussions and guide eligibility for clinical trials.

## Methods

### Patients

Information about patients with SBNETs (site codes: C17.1, C17.2, and C17.3; histologic codes: 8013, 8150 to 8156, 8240 to 8247, 8249, and 9091) diagnosed between 1988 and 2014 according to the International Classification of Disease for Oncology, third edition (ICD-O-3), was obtained from the SEER database, a national cancer registry managed by the United States National Cancer Institute, which collected information related to sociodemography and clinicopathology. Parameters included race, age, year of diagnosis, sex, tumor site and size, tumor extension, tumor differentiation, regional LN removal, regional LN involvement, distant metastasis, surgery, and survival status. Tumor size and extension were used to determine T categories according to the AJCC eighth edition stage classification system. Tumor grade according to the World Health Organization (WHO) classification was not available in the SEER database, only tumor differentiation was retrieved. Eligibility criteria were as follows: SBNETs were the first and only malignant tumor; patient is 18 years of age or older; surgical resection was performed; there is complete LN information; and the survival time is at least one month. Cause-specific survival (CSS) was the primary outcome measure.

### Statistical Analysis

Descriptive statistics was used to report the basic clinicopathological characteristics of the patients for total study population and by each diagnosis-year cohort (1988–1999 vs. 2000–2014). The cutoff points of the RLNs, NLNs, LNR, and LODDS were determined by the X-tile program by using the minimum *P*-values from log-rank chi-square analysis in terms of CSS (22). Survival analyses were performed using Kaplan–Meier curves with log-rank test. Logistic regression analysis was used to evaluate the influence of the different periods. Univariate Cox proportional hazards regression was used to identify potential prognostic factors. Multivariable Cox regression models were built to jointly assess the prognostic ability of the different LN staging schemes and other potential prognostic indicators. The Akaike information criterion (AIC) and the Harrell consistency index (HCI) were used to assess the relative discriminative power of different LN staging systems. An HCI of 0.5 indicated no predictive power, and an HCI of 1 indicated complete differentiation (23). In general, a predictive model with a high HCI indicated a better discriminating ability, while a low AIC indicated a better model fit (24). All analyses were carried out with SPSS version 22.0 and R version 3.6.1. For all analysis, *P* <0.05 was considered significant, and all tests were two-tailed.

## Result

### Patient Characteristics

A total of 3,680 qualified participants with SBNETs diagnosed from 1988 to 2014 in the SEER database were enrolled in this study. The program selection details for the SEER database queries were shown in Supplementary Figure 1. The clinicopathological characteristics and distributions of different LN staging systems for the study population were shown in Table 1. The total study population consisted of 1,814 males (49.5%) and 1,866 females (50.7%). The median age at diagnosis was 59.0 years (range, 18–100 years). The median follow-up time was 53.0 months (range, 1–318 months). Overall, 304 (8.3%) SBNETs were located in the duodenum, 3,064 (83.3%) in the ileum, and 312 (8.5%) in the jejunum. According to the AJCC eighth edition staging criteria, patients with T4 disease (43.0%) were the most numerous, followed by patients with T1 disease (9.3%), T2 disease (19.7%), and T3 disease (27.9%). There were 2,963 (80.5%) patients with SBNETs who had LN metastases and 717 (19.5%) patients with no LN metastases. The median numbers of PLNs, RLNs, NLNs, LNR, and LODDS in the whole cohort were 2, 12, 7, 0.23, and −1.18, respectively.

**Table 1:** Demographics and clinicopathological characteristics of patients with SBNETs.

**Characteristic**		**Patients (%)**
**Race**		
	White	3,181 (86.4%)
	Black	416 (11.3%)
	Others	83 (2.3%)
**Year of diagnosis**		
	1988–1999	424 (11.5%)
	2000–2014	3,256 (88.5%)
**Sex**		
	Male	1,814 (49.3%)
	Female	1,866 (50.7%)
**Age**		
	≤ 60	1,954 (53.1%)
	>60	1,726 (46.9%)
**Tumor site**		
	Duodenum	304 (8.3%)
	Ileum	3,064 (83.3%)
	Jejunum	312 (8.5%)
**Tumor size**		
	≤ 1 cm	655 (17.8%)
	≤ 2 cm	1,393 (37.9%)
	≤ 4 cm	1,176 (32.0%)
	>4 cm	243 (6.6%)
	Unknown	203 (5.8%)
**Tumor differentiation**		
	Well differentiated	1,562 (42.4%)
	Moderately differentiated	385 (10.5%)
	Poorly differentiated	61 (1.7%)
	Undifferentiated	11 (0.3%)
	Unknown	1,661 (45.1%)
**T stage**		
	T1	342 (9.3%)
	T2	726 (19.7%)
	T3	1,028 (27.9%)
	T4	1,584 (43.0%)
**N category**		
	Node negative	717 (19.5%)
	Node positive	2,963 (80.5%)
**M stage**		
	M0	2,682 (72.9%)
	M1	998 (27.1%)
PLNs	Median	2
RLNs	Median	12
NLNs	Median	7
LNR	Median	0.23
LODDS	Median	−1.18
**RLNs**		
	≤ 11	1,990 (54.1%)
	>11	1,690 (45.9%)
**NLNs**		
	≤ 7	1,908 (51.8%)
	>7	1,772 (48.2%)
**LNR**		
	0	734 (19.9%)
	≤ 0.4	1,854 (50.4%)
	>0.4	1,092 (29.7%)
**LODDS**		
	≤ −1.3	1,739 (47.3%)
	−1.3 to −0.3	899 (24.4%)
	> −0.3	1,042 (28.3%)

*PLNs, positive lymph nodes; RLNs, resected lymph nodes; NLNs, negative lymph nodes; LNR, lymph node ratio; LODDS, the log odds of positive lymph nodes*.

### Characteristics of the LN Staging Schemes

The threshold of PLN was determined by the AJCC eighth edition staging system. The X-tile software was used to perform log-rank chi-square analysis to estimate the cutoff value of RLNs, NLNs, LNR, and LODDS with the minimum *P*-values. For the RLN staging system, we used 11 as the best cutoff value and divided the RLNs into two groups as follows: RLNs ≤ 11 and RLNs > 11 (Supplementary Figure 2). For the NLN staging system, we used seven as the best cutoff value and divided the NLNs into two groups as follows: NLNs ≤ 7 and NLNs > 7 (Supplementary Figure 3). For the LNR staging system, we used 0 and 0.4 as the best cutoff values and divided the LNR into three groups as follows: LNR = 0; LNR ≤ 0.04; and LNR > 0.4 (Supplementary Figure 4). For the LODDS staging system, we used −1.3 and −0.3 as the best cutoff values and divided the LODDS into three groups as follows: LODDS ≤ −1.35; −1.35 < LODDS ≤ −0.3; and LODDS > −0.3 (Supplementary Figure 5).

### Survival

**Figure 1:**
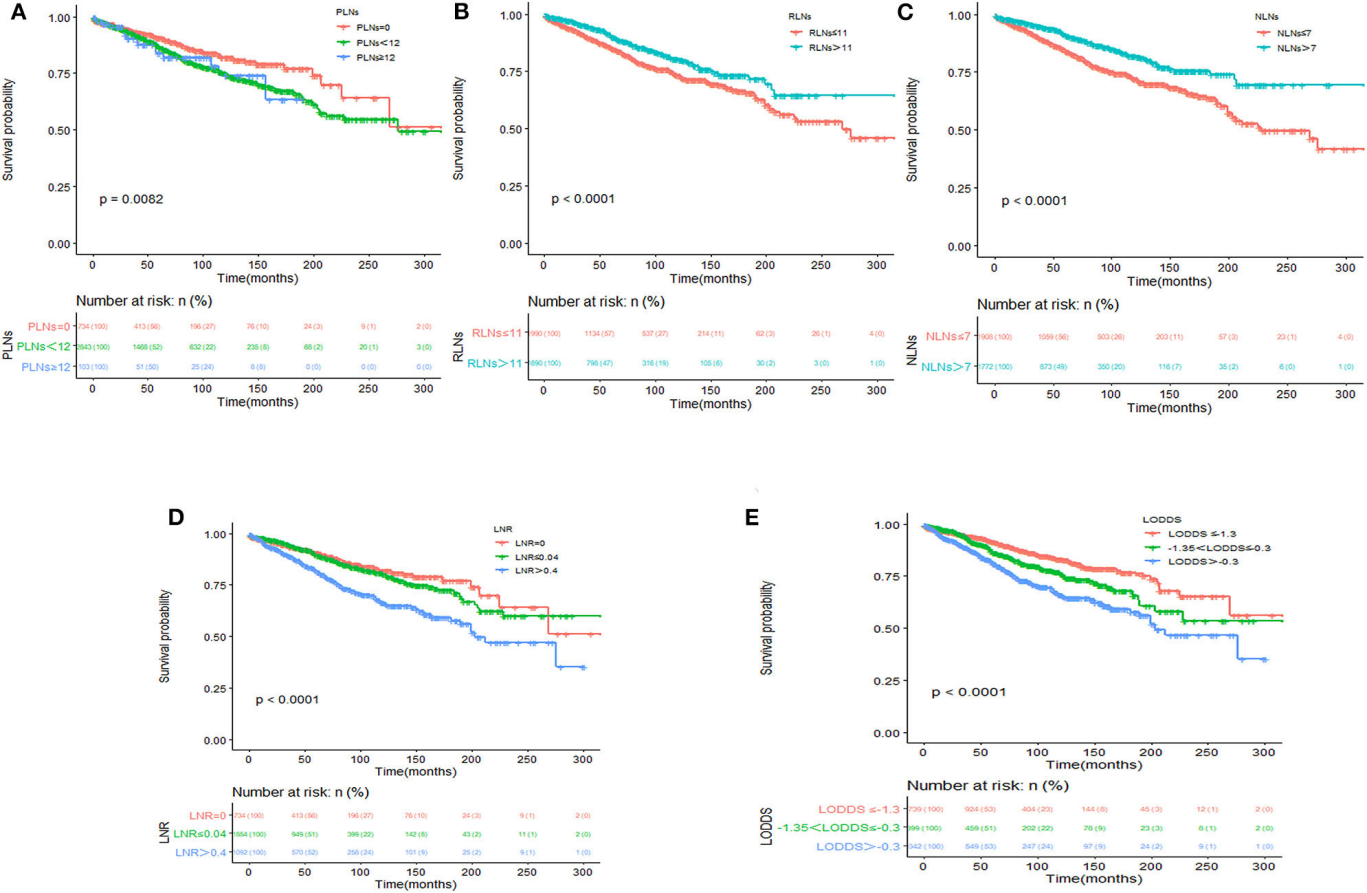
Kaplan–Meier survival analysis according to PLN classification **(A)**, RLN classification **(B)**, NLN classification **(C)**, LNR classification **(D)**, and LODDS classification **(E)** for cause-specific survival in patients with SBNETs.

**Figure 2:**
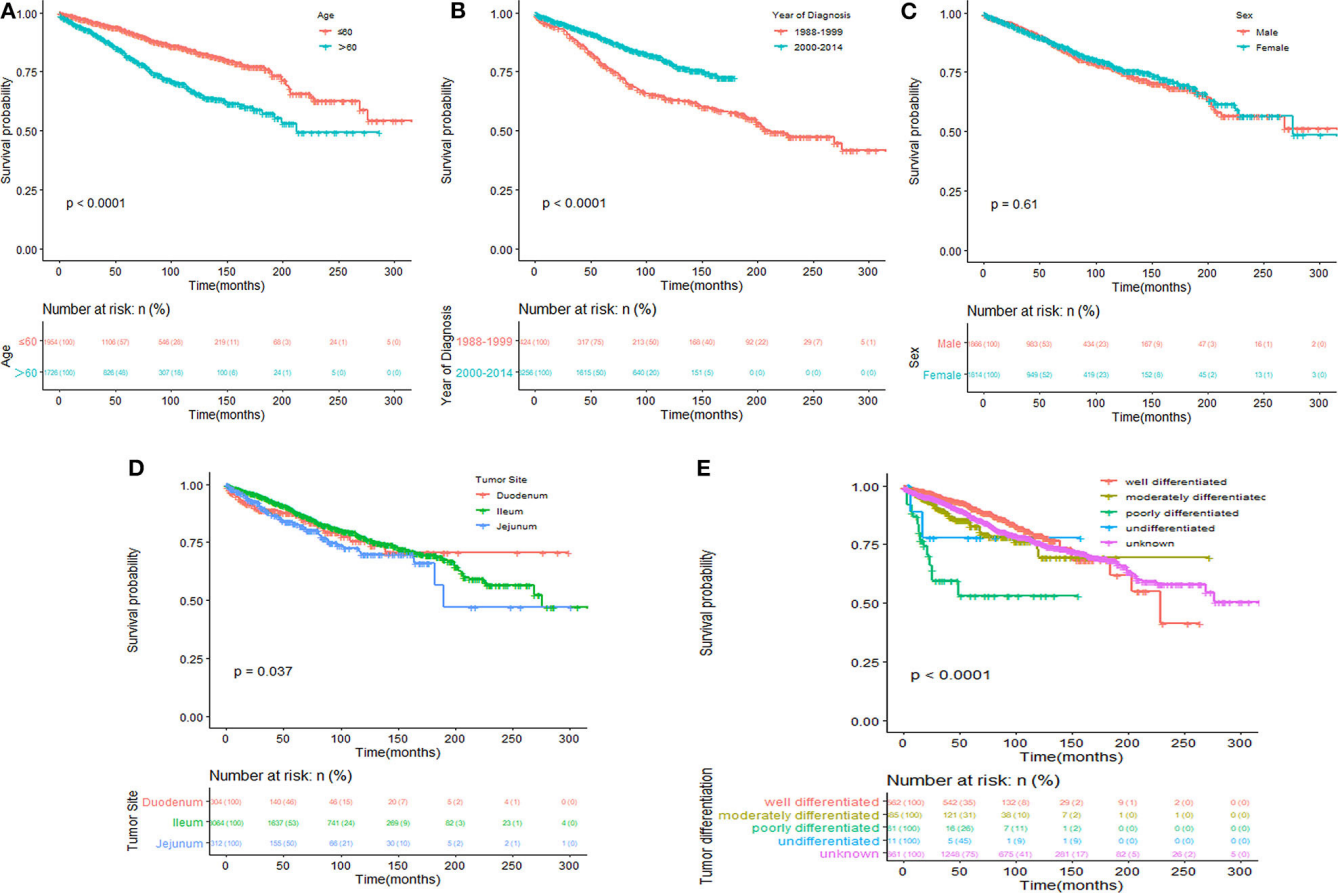
Kaplan–Meier survival analysis according to age **(A)**, year of diagnosis **(B)**, sex **(C)**, tumor site **(D)**, and tumor differentiation **(E)** for cause-specific survival in patients with SBNETs.

Kaplan–Meier survival curves and survival data based on different LN staging systems were shown in Figure 1 for all patients. The 5-year CSS of patients without LN involvement was 90.8%, with 86.7% for with PLNs <12 and with 83.9% for those with PLNs ≥ 12 (*P* <0.001; Figure 1A). The 5-year CSS was 85.3% for patients with RLNs ≤ 11 and 90.3% for those with RLNs > 11 (*P* <0.001; Figure 1B). The 5-year CSS was 84.5% for patients with NLNs ≤ 7 and 91.1% for those with NLNs > 7 (*P* <0.001; Figure 1C). The 5-year CSS rates were 90.5, 89.6, and 81.8% for patients in the LNR = 0, LNR ≤ 0.4, and LNR > 0.4 subgroups, respectively (*P* <0.001; Figure 1D). The 5-year CSS rates were 91.6, 86.9, and 81.5% in the LODDS ≤ −1.3, −1.3 < LODDS ≤ −0.3, and LODDS > −0.3 subgroups, respectively (*P* <0.001; Figure 1E). These survival disparities remained among the subsets of age (*P* <0.001; Figure 2A), tumor site (*P* <0.001; Figure 2D), and tumor differentiation (*P* <0.001; Figure 2E) but not in the subsets of sex (*P* = 0.61; Figure 2C). When stratified by year of diagnosis, CSS improved significantly over time (*P* <0.001; Figure 2B).

### Disparities Between Diagnosis-Year Cohorts

**Figure 3:**
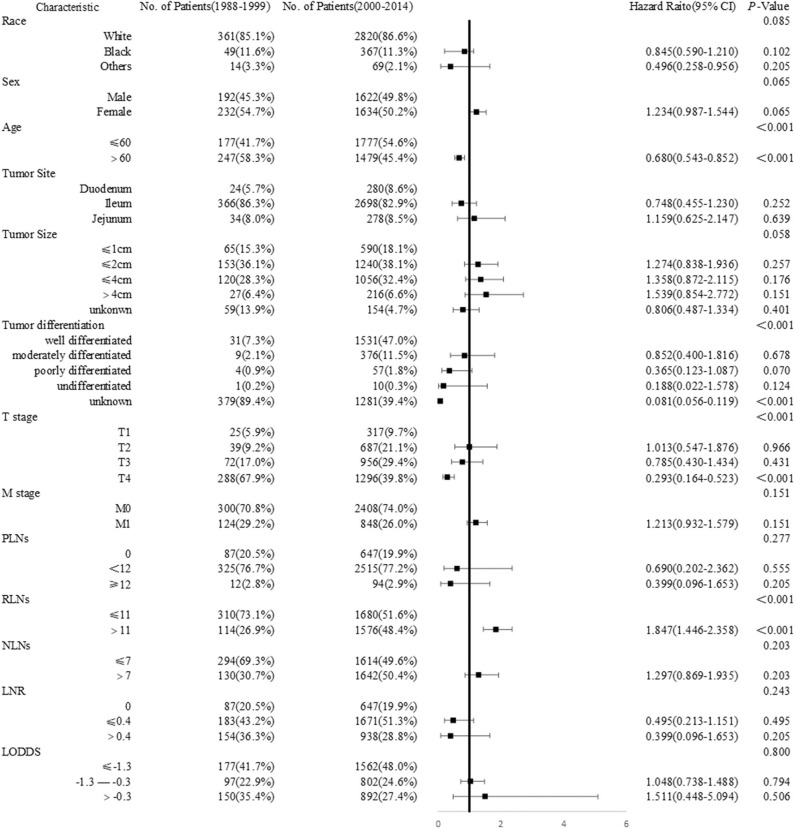
Forest plot showing the logistic regression analyses of the difference between years of diagnosis.

To further explore the period-specific differences observed, we performed additional analysis of diagnosis-year cohorts among patients with SBNETs. These significant differences over time were seen in age (*P* <0.001), tumor differentiation (*P* <0.001), T stage(*P* <0.001), and RLNs (*P* <0.001) subgroups, except in race, sex, tumor site, M stage, PLNs, NLNs, and LNR subgroups (Figure 3). The most common age range at initial diagnosis was ≤ 60 years in the 2000–2014 cohort, whereas it was >60 years in the 1988–1999 cohort (HR 0.680, 95% CI 0.543–0.852, *P* <0.001). Patients with SBNETs were more likely to have advanced T disease and RLNs ≤ 11 in the 1988–1999 cohort, whereas they were likely to have early T disease and RLNs > 11 in the 2000–2014 cohort.

### Prognostic Abilities of Different LN Staging Systems in the SEER Database

Univariate Cox survival analysis indicated that the age, year of diagnosis, tumor site, tumor size, tumor differentiation, T stage, M stage, and the number of PLNs, NLNs, RLNs, LNR, and LODDS were closely related to CSS of SBNETs (Table 2). Multivariate Cox analysis was used to assess the independent prognosis of the different LN staging systems separately. Higher numbers of RLNs (HR: 0.729, 95% CI: 0.605–0.878, *P* <0.001; Supplementary Table 1) and NLNs (HR: 0.689, 95% CI: 0.574–0.828, *P* <0.001; Supplementary Table 2) were related to a better CSS. But the number of PLNs was not prognostic for CSS (PLNs = 0 as the reference; PLNs <12: HR: 1.124, 95% CI: 0.885–1.427, *P* = 0.338; PLNs ≥ 12: HR: 0.912, 95% CI: 0.549–1.517, *P* = 0.724; Supplementary Table 3). The LNR (LNR = 0 as the reference; LNR ≤ 0.4: HR: 0.952, 95% CI: 0.737–1.230, *P* = 0.709; LNR > 0.4: HR: 1.352, 95% CI: 1.047–1.746, *P* = 0.021; Table 3) and LODDS (LODDS ≤ −1.3 as the reference; −1.3 < LODDS ≤ −0.3: HR: 1.048, 95% CI: 0.832–1.320, *P* = 0.693; LODDS > −0.3: HR: 1.490, 95% CI: 1.221–1.818, *P* <0.001; Table 4) persisted as significant and independent prognostic factors of CSS.

**Table 2:** Univariate analysis of prognostic factors influencing CCS in patients with SBNETs.

**Characteristic**		**HR (95% CI)**	***P*-value**
**Race**			0.305
	White		
	Black	0.797 (0.590–1.075)	0.137
	Others	0.872 (0.480–1.585)	0.653
**Year of diagnosis**			<0.001
	1988–1999		
	2000–2014	0.530 (0.436–0.644)	<0.001
**Sex**			0.606
	Male		
	Female	1.022 (0.940–1.112)	0.606
**Age**			<0.001
	≤ 60		
	>60	2.189 (1.840–2.603)	<0.001
**Tumor site**			0.037
	Duodenum		
	Ileum	0.825 (0.607–1.121)	0.219
	Jejunum	1.147 (0.777–1.693)	0.491
**Tumor size**			<0.001
	≤ 1 cm		
	≤ 2 cm	2.075 (1.471–2.928)	<0.001
	≤ 4 cm	3.074 (2.188–4.318)	<0.001
	>4 cm	4.927 (3.314–7.324)	<0.001
	Unknown	4.532 (3.075–6.680)	<0.001
**Tumor differentiation**			<0.001
	Well differentiated		
	Moderately differentiated	1.728 (1.235–2.417)	0.001
	Poorly differentiated	5.704 (3.677–8.848)	<0.001
	Undifferentiated	2.297 (0.568–9.294)	0.244
	Unknown	1.336 (1.078–1.657)	0.008
**T stage**			<0.001
	T1		
	T2	1.860 (0.853–4.057)	0.119
	T3	4.999 (2.435–10.265)	<0.001
	T4	10.668 (5.299–21.480)	<0.001
**N stage**			<0.001
	Node negative		
	Node positive	1.497 (1.180–1.898)	0.001
**M stage**			<0.001
	M0		
	M1	3.796 (3.204–4.496)	<0.001
**PLNs**			0.008
	0		
	<12	1.434 (1.138–1.807)	0.002
	≥12	1.489 (0.873–2.540)	0.144
**RLNs**			<0.001
	≤ 11		
	>11	0.661 (0.552–0.792)	<0.001
**NLNs**			<0.001
	≤ 7		
	>7	0.580 (0.484–0.694)	<0.001
**LNR**			0.001
	0		
	≤ 0.4	1.107 (0.863–1.421)	0.424
	>0.4	1.979 (1.546–2.533)	<0.001
**LODDS**			<0.001
	≤ −1.3		
	−1.3 to −0.3	1.397 (1.115–1.750)	0.004
	> −0.3	2.116 (1.743–2.569)	<0.001

*HR, hazard ratio; CI, confidence interval; PLNs, positive lymph nodes; RLNs, resected lymph nodes; NLNs, negative lymph nodes; LNR, lymph node ratio; LODDS, the log odds of positive lymph nodes*.

**Table 3:** Multivariate analysis of LNR influencing CCS in patients with SBNETs.

**Characteristic**		**HR (95% CI)**	***P*-value**
**Race**			0.833
	White		
	Black	0.999 (0.735–1.357)	0.993
	Others	0.828 (0.449–1.527)	0.546
**Year of diagnosis**			<0.001
	1988–1999		
	2000–2014	0.631 (0.512–0.776)	<0.001
**Sex**			0.681
	Male		
	Female	1.037 (0.873–1.230)	0.681
**Age**			<0.001
	≤ 60		
	>60	2.192 (1.837–2.617)	<0.001
**Tumor site**			<0.001
	Duodenum		
	Ileum	0.475 (0.344–0.657)	<0.001
	Jejunum	0.533 (0.355–0.802)	0.003
**Tumor size**			0.013
	≤ 1 cm		
	≤ 2 cm	1.145 (0.780–1.681)	0.489
	≤ 4 cm	1.506 (0.972–2.333)	0.067
	>4 cm	1.514 (0.992–2.312)	0.055
	Unknown		
**Tumor differentiation**			<0.001
	Well differentiated		
	Moderately differentiated	1.803 (1.286–2.528)	0.001
	Poorly differentiated	4.169 (2.670–6.510)	<0.001
	Undifferentiated	0.932 (0.229–3.793)	0.922
	Unknown	1.226 (0.982–1.531)	0.072
**T stage**			<0.001
	T1		
	T2	1.593 (0.689–3.682)	0.276
	T3	3.607 (1.611–8.078)	0.002
	T4	5.076 (2.296–11.223)	<0.001
**M stage**			<0.001
	M0		
	M1	2.829 (2.360–3.391)	<0.001
**LNR**			<0.001
	0		
	≤ 0.4	0.952 (0.737–1.230)	0.709
	>0.4	1.352 (1.047–1.746)	0.021

*HR, hazard ratio; CI, confidence interval; LNR, lymph node ratio*.

**Table 4:** Multivariate analysis of LODDS influencing CCS in patients with SBNETs.

**Characteristic**		**HR (95% CI)**	***P*-value**
**Race**			0.878
	White		
	Black	0.994 (0.732–1.351)	0.970
	Others	0.853 (0.463–1.572)	0.610
**Year of diagnosis**			<0.001
	1988–1999		
	2000–2014	0.629 (0.511–0.774)	<0.001
**Sex**			0.676
	Male		
	Female	1.037 (0.874–1.231)	0.676
**Age**			<0.001
	≤ 60		
	>60	2.205 (1.848–2.632)	<0.001
**Tumor site**			<0.001
	Duodenum		
	Ileum	0.473 (0.343–0.652)	<0.001
	Jejunum	0.536 (0.357–0.805)	0.003
**Tumor size**			0.011
	≤ 1 cm		
	≤ 2 cm	1.002 (0.684–1.468)	0.992
	≤ 4 cm	1.138 (0.775–1.671)	0.509
	>4 cm	1.506 (0.973–2.333)	0.066
	Unknown	1.514 (0.992–2.310)	0.055
**Tumor differentiation**			<0.001
	Well differentiated		
	Moderately differentiated	1.782 (1.271–2.499)	0.001
	Poorly differentiated	4.137 (2.649–6.461)	<0.001
	Undifferentiated	1.076 (0.264–4.388)	0.918
	Unknown	1.217 (0.974–1.520)	0.084
**T stage**			<0.001
	T1		
	T2	1.557 (0.674–3.598)	0.300
	T3	3.474 (1.554–7.766)	0.002
	T4	4.883 (2.211–10.784)	<0.001
**M stage**			<0.001
	M0		
	M1	2.846 (2.375–3.411)	<0.001
**LODDS**			<0.001
	≤ −1.3		
	−1.3 to −0.3	1.048 (0.832–1.320)	0.693
	> −0.3	1.490 (1.221–1.818)	<0.001

*HR, hazard ratio; CI, confidence interval; LODDS, the log odds of positive lymph nodes*.

### Comparison of Prognostic Values of the Different LN Staging Systems

**Figure 4:**
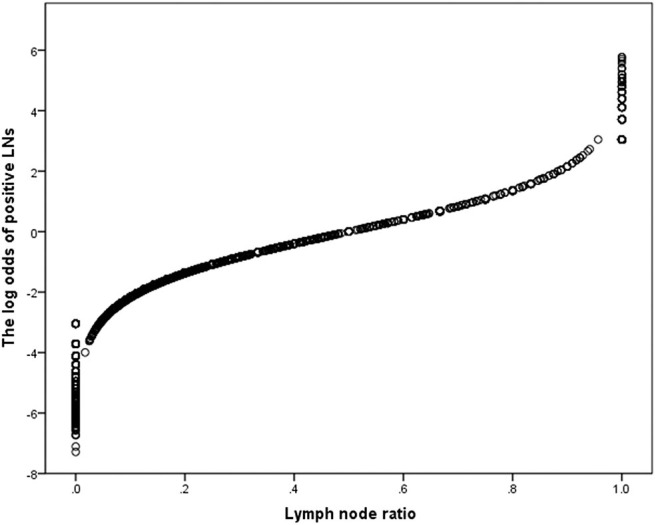
Scatter plot presenting the distribution of LODDS and LNR.

The LN status was evaluated as a categorical variable to analyze the prognostic discriminating power of different LN staging systems, after controlling for age, year of diagnosis, tumor size, primary tumor site, tumor differentiation, T stage, and M stage data. In the whole population, LODDS staging systems showed the best prognostic performance (HCI: 0.766, AIC: 7,575.154; Supplementary Table 4). To assess whether the ability of the predicted prognosis of different LN staging systems was affected by artificially determined cutoff values, the LN status was modeled as a continuous variable for repeated analysis. The LNR staging system had better prognostic performances (HCI: 0.766, AIC: 7,578.546; Supplementary Table 4). However, tumor differentiation was not available for approximately half of the analyzed population, and 5.8% of the patients had incomplete data for tumor size. An additional analysis was constructed that eliminated these missing data. In this sensitivity model, LODDS still had better prognostic performance (HCI: 0.795, AIC: 2,157.289; Table 5) in the categorical cohort, whereas the LNR staging system had better prognostic performance in the continuous cohort (HCI: 0.794, AIC: 2,161.076). Further analysis based on different RLNs showed that when the RLNs were insufficient (RLNs ≤ 11), the LODDS (HCI: 0.769, AIC: 1,088.731) staging system maintained the best prognostic performance as well as when the RLNs were >11 (LODDS, HCI: 0.835, AIC: 825.692). Then scatter plots were created to evaluate the relationship between LODDS and LNR (Figure 4). The correlation between LNR and LODDS was near, but not completely linear. When patients have different LNR, the LODDS has a one-to-one mapping value for each LNR, and as the LNR increases, the value of LODDS also increases.

**Table 5:** Prognostic performance of different lymph node staging systems by stratifying the number of RLNs after eliminating the missing data.

**Variables**	**Patients(*****N*** **=** **1,959)**		**RLN** **≤** **11**		**RLNs** **>** **11**	
	**C-index (95% CI)**	**AIC**	**C-index (95% CI)**	**AIC**	**C-index (95% CI)**	**AIC**
PLNs (continuous)	0.791 (0.753–0.828)	2,163.240	0.768 (0.713–0.824)	1,089.694	0.829 (0.785–0.874)	827.949
NLNs (continuous)	0.793 (0.755–0.830)	2,162.014	0.762 (0.705–0.819)	1,089.889	0.834 (0.790–0.878)	830.529
RLNs (continuous)	0.793 (0.755–0.830)	2,164.058	–	–	–	–
LNR (continuous)	0.794 (0.757–0.830)	2,161.076	0.766 (0.710–0.819)	1,089.631	0.832 (0.788–0.875)	828.381
LODDS (continuous)	0.792 (0.754–0.829)	2,162.188	0.765 (0.709–0.821)	1,089.988	0.831 (0.786–0.876)	829.105
PLNs	0.792 (0.754–0.830)	2,164.943	0.761 (0.704–0.818)	1,090.623	0.832 (0.787–0.877)	830.453
NLNs	0.793 (0.757–0.830)	2,162.611	0.765 (0.708–0.821)	1,090.761	0.834 (0.793–0.875)	829.288
RLNs	0.793 (0.756–0.830)	2,163.292	–	–	–	–
LNR	0.793 (0.756–0.831)	2,163.453	0.767 (0.712–0.822)	1,090.050	0.834 (0.791–0.878)	830.355
LODDS	0.795 (0.759–0.831)	2,157.289	0.769 (0.714–0.877)	1,088.731	0.835 (0.788–0.875)	825.692

*CI, confidence interval; PLNs, positive lymph nodes; RLNs, resected lymph nodes; NLNs, negative lymph nodes; LNR, lymph node ratio; LODDS, the log odds of positive lymph nodes*.

## Discussion

SBNETs are a heterogeneous group of tumors with a wide spectrum of clinical features and different clinical outcomes (25). Regional LN involvement is one of the main metastatic patterns and the most important prognostic parameter of SBNETs (26). Therefore, accurate staging of LN status can more accurately predict cancer risk and develop postoperative treatment options and surveillance for patients with SBNETs (27). Given this, several alternative staging systems have been proposed to address the shortcomings of the LN classification of the AJCC staging system. Some researchers have evaluated different staging systems with mixed results, supporting the prognostic ability of the LNR staging system (28, 29). In the current study, we first used population-based data to assess the relative discriminative ability of different LN staging systems to predict CSS in patients with SBNETs. We found that LN status stratified by the numbers of RLNs, NLNs, LNR, and LODDS predicted CSS in patients of SBNETs, while the number of PLNs failed. After the categorization based on the data of the current study, the LODDS classification showed a better predictive value.

The importance of LN involvement in determining the prognosis of patients with SBNETs has been demonstrated (29). Our data confirm the important prognostic role of LN status in patients with SBNETs. All LN staging systems stratified patients progressively based on CSS. However, patients with PLNs <12 vs. PLNs ≥ 12 had overlapping survival curves. These findings suggested that the use of PLNs to define pN classification may had some prognostic deficiencies when patients were stratified by specific categorical cutoff values, as reported in a previous study (27).

Several studies had noted that a minimal number of LNs need to be evaluated for accurate stage. For example, Zaidi et al. (30) had advocated that accurate LN staging requires a minimum of eight LNs for examination. In this study, the optimal cut-point of RLNs was 11. A higher number of RLNs was associated with longer CSS. In view of this, we conducted subgroup studies based on different RLNs to analyze the prognostic accuracy of each LN staging system. The PLN staging systems performed relatively poorly when the RLN was insufficient (RLNs ≤ 11), whereas when RLN was >11, the PLN staging systems had a better prognostic value. This had shown that the PLN was significantly correlated with the RLN, especially when the RLN was insufficient, which may cause the missed PLNs, resulting in staging migration (31, 32).

LNR was proposed as a mean to take the total number of LN into account when evaluating LN status to avoid the stage migration phenomenon (33, 34). We noted that LNR was the best-performing model among patients when examined as a continuous variable, regardless of how many LNs were evaluated. However, for patients with either a very low or high LNR, the prognostic discriminatory ability of LNR seemed poor. These findings may be somewhat intuitive, since most clinicians would agree that a patient with one RLN and one NLN has a different prognosis compared with a patient with 12 RLNs and 12 NLNs, even though both patients have an LNR score of 1.

Although LODDS has been previously examined in a large cohort of patients with other cancers, the role of LODDS among patients with SBNETs has not been well-studied. In the present study, we noted that the heterogeneity of LODDS in patients with LNR scores of 0 or 1 indicated that the LODDS has the potential to distinguish patients with the same LNR classification but different prognosis, especially if those LNRs were 0 and 1.

Notably, we also found that LODDS had the highest discrimination in patients who had <12 RLNs. Collectively, these data strongly suggested that LODDS should be the preferred LN staging systems to stratify patients when the RLN was insufficient. Although a more complicated calculation than LNR, LODDS seems to be the most reliable method of patient stratification in N classification.

Several studies indicated that tumor grade may significantly impact the prognosis of SBNETs (35, 36). The current widely used histological grading system for NETs was firstly proposed by ENETS and endorsed by WHO in 2010 (fourth edition) (37, 38). In the SEER database, tumor grade according to the WHO 2010 classification was not available, only tumor differentiation was retrieved. This grading classification based on tumor proliferative activity was assessed by mitotic count and Ki-67 index (38). It has been revised in 2017 for pancreatic neuroendocrine neoplasms, and the fifth edition of the WHO classification of tumors of the digestive system was in press in 2019 (39). The essential change concerned the recognition that well-differentiated NETs may be of high grade, but these neoplasms remained well-differentiated genetically and distinguished from poorly differentiated neuroendocrine carcinoma. Supplementary Table 5 showed the key points of the grading scheme. Nevertheless, the cutoff level of the Ki-67 index differentiating G1 and G2 diseases was still an open issue. Strosberg et al. (36) and Khan et al. (40) suggested that the threshold to classify G1 and G2 should be revised from 2 to 5% in SBNET patients. On the other hand, Cunningham et al. found a cutoff of 1% to be more accurate than a cutoff of 2% in a study of metastatic SBNETs (41). Future studies possibly performed by additional and larger patient cohorts may contribute important data on this topic.

Regional lymphadenopathy was associated with desmoplasia and fibrosis of the mesentery forming a mesenteric mass, which may impact on clinical and staging features in SBNETs (42–44). The updated AJCC 8th edition had incorporated mesenteric masses in staging for midgut NETs, but the prognostic validity of this classification has yet to be validated. In a study of 72 cases of SBNET resections, Gonzalez et al. (45) demonstrated that the presence of a mesenteric mass was significantly associated with lymph nodal and liver metastasis, lymphovascular invasion, T3 or T4 disease, and increased disease progression and death. Malik et al. (46) also found that patients with a mesenteric mass were more likely to have advanced T status and confirmed that mesenteric involvement represented more extensive disease and was also associated with more aggressive treatment. If feasible, surgery was required for the primary tumor, regional LNs, and mesenteric masses. However, large mesenteric masses or encasement of the proximal mesenteric artery and vein may prevent safe resection (47).

This study had several limitations. First, it was a retrospective study based on the SEER database, so there were inevitably some selection biases. The SEER database lacked some clinical information such as vascular invasion, mesenteric mass, and specific locations of LN metastasis. Tumor grade according to WHO classification was not available in the SEER database. However, our findings are credible and widely applicable because the SEER database was based on the US population and clinical practice. Secondly, classification systems had changed in use during the long period and may have potentially exceeded high heterogeneity of the SBNETs. Nevertheless, although age, tumor differentiation, T status, and RLN showed disparities between diagnosis-year cohorts, LODDS showed no significant difference, indicating that the LODDS staging system had excellent applicability even if the period is long. Additionally, the disease-free survival and molecular pathologic characteristics were not available in this study, which may result in a limitation on the survival and LN metastasis analysis.

## Conclusion

In conclusion, the current study had demonstrated that LODDS and LNR were more useful and powerful than the PLN classification for prognostic assessment for SBNETs. It should be recommended that LODDS was a better predicator of survival when LN status was stratified as a categorical variable, especially in patients with either a very low or high LNR. As such, LODDS should be considered when assessing the prognosis of patients with SBNETs to allow a more reliable means to stratify patient survival.

## Data Availability Statement

Publicly available datasets were analyzed in this study. This data can be found here: https://seer.cancer.gov/data/.

## Author Contributions

YY and SJ conceived the concept and designed the study. SJ and LZ collected and analyzed the data. HS and CX prepared the figures. YY and SJ wrote the paper. All authors reviewed and approved the manuscript.

## Conflict of Interest

The authors declare that the research was conducted in the absence of any commercial or financial relationships that could be construed as a potential conflict of interest.

## Abbreviations

LN: lymph node
SBNETs: small bowel neuroendocrine tumors
SEER, Surveillance: Epidemiology and End Results
PLNs: positive lymph nodes
RLNs: resected lymph nodes
NLNs: negative lymph nodes
LNR: lymph node ratio
LODDS: the log odds of positive lymph nodes
CSS: cause-specific survival
AIC: Akaike information criterion
HCI: Harrell consistency index
PLNs: positive lymph nodes
ENETS: the European Neuroendocrine Tumor Society
AJCC: the American Joint Committee on Cancer
ICD-O-3, the International Classification of Disease for Oncology: third edition
WHO: World Health Organization
HR: hazard ratio
CI: confidence interval.
