# Supplementary material for: Prognostic Performance of Different Lymph Node Staging Systems in Patients With Small Bowel Neuroendocrine Tumors
Source: Front Endocrinol (Lausanne). 2020 Jul 7;11:402. doi: 10.3389/fendo.2020.00402 (PMC7358303; doi:10.3389/fendo.2020.00402)
Supplement: Supplementary file 1 [file Table_1.docx]

**Supplementary Tables**

Supplementary Table 1 Multivariate analysis of RLNs influencing CCS in patients with SBNETs

| Characteristic | | HR (95%CI) | *P* value |
| --- | --- | --- | --- |
| Race |  |  | 0.818 |
|  | White |  |  |
|  | Black | 1.004（0.738-1.365） | 0.977 |
|  | Others | 0.821（0.446-1.514） | 0.528 |
| Year of Diagnosis |  |  | ＜0.001 |
|  | 1988-1999 |  |  |
|  | 2000-2014 | 0.650（0.527-0.801） | ＜0.001 |
| Sex |  |  | 0.713 |
|  | Male |  |  |
|  | Female | 1.033（0.870-1.226） | 0.713 |
| Age |  |  | ＜0.001 |
|  | ≤60 |  |  |
|  | ＞60 | 2.148（1.800-2.564） | ＜0.001 |
| Tumor Site |  |  | ＜0.001 |
|  | Duodenum |  |  |
|  | Ileum | 0.514（0.372-0.711） | ＜0.001 |
|  | Jejunum | 0.581（0.387-0.873） | 0.009 |
| Tumor Size |  |  | 0.011 |
|  | ≤1cm |  |  |
|  | ≤2cm | 1.045（0.714-1.529） | 0.822 |
|  | ≤4cm | 1.232（0.841-1.805） | 0.284 |
|  | ＞4cm | 1.587（1.025-2.457） | 0.038 |
|  | unkonwn | 1.556（1.020-2.375） | 0.040 |
| Tumor differentiation |  |  | ＜0.001 |
|  | well differentiated |  |  |
|  | moderately differentiated | 1.827（1.303-2.561） | ＜0.001 |
|  | poorly differentiated | 4.138（2.652-6.459） | ＜0.001 |
|  | undifferentiated | 1.021（0.251-4.165） | 0.976 |
|  | unknown | 1.185（0.948-1.418） | 0.137 |
| T stage |  |  | ＜0.001 |
|  | T1 |  |  |
|  | T2 | 1.617（0.701-3.731） | 0.260 |
|  | T3 | 3.733（1.673-8.330） | 0.001 |
|  | T4 | 5.294（2.403-11.662） | ＜0.001 |
| M stage |  |  | ＜0.001 |
|  | M0 |  |  |
|  | M1 | 2.875（2.399-3.445） | ＜0.001 |
| RLNs |  |  | ＜0.001 |
|  | ≤11 |  |  |
|  | ＞11 | 0.729（0.605-0.878） | ＜0.001 |

HR, hazard ratio; CI, confidence interval; RLNs, resected lymph nodes.

Supplementary Table 2 Multivariate analysis of NLNs influencing CCS in patients with SBNETs

| Characteristic | | HR (95%CI) | *P* value |
| --- | --- | --- | --- |
| Race |  |  | 0.771 |
|  | White |  |  |
|  | Black | 1.008（0.742-1.369） | 0.961 |
|  | Others | 0.800（0.434-1.475） | 0.475 |
| Year of Diagnosis |  |  | ＜0.001 |
|  | 1988-1999 |  |  |
|  | 2000-2014 | 0.649（0.527-0.800） | ＜0.001 |
| Sex |  |  | 0.856 |
|  | Male |  |  |
|  | Female | 1.016（0.856-1.206） | 0.856 |
| Age |  |  | ＜0.001 |
|  | ≤60 |  |  |
|  | ＞60 | 2.158（1.808-2.576） | ＜0.001 |
| Tumor Site |  |  | ＜0.001 |
|  | Duodenum |  |  |
|  | Ileum | 0.510（0.369-0.704） | ＜0.001 |
|  | Jejunum | 0.572（0.381-0.860） | 0.007 |
| Tumor Size |  |  | 0.009 |
|  | ≤1cm |  |  |
|  | ≤2cm | 1.053（0.719-1.514） | 0.791 |
|  | ≤4cm | 1.225（0.836-1.794） | 0.297 |
|  | ＞4cm | 1.622（1.048-2.512） | 0.030 |
|  | unkonwn | 1.559（1.022-2.379） | 0.039 |
| Tumor differentiation |  |  | ＜0.001 |
|  | well differentiated |  |  |
|  | moderately differentiated | 1.804（1.287-2.529） | 0.001 |
|  | poorly differentiated | 4.071（2.608-6.355） | ＜0.001 |
|  | undifferentiated | 1.115（0.274-4.547） | 0.879 |
|  | unknown | 1.196（0.958-1.495） | 0.114 |
| T stage |  |  | ＜0.001 |
|  | T1 |  |  |
|  | T2 | 1.590（0.689-3.672） | 0.277 |
|  | T3 | 3.666（1.643-8.183） | 0.002 |
|  | T4 | 5.189（2.355-11.433） | ＜0.001 |
| M stage |  |  | ＜0.001 |
|  | M0 |  |  |
|  | M1 | 2.848（2.376-3.414） | ＜0.001 |
| NLNs |  |  | ＜0.001 |
|  | ≤7 |  |  |
|  | ＞7 | 0.689（0.574-0.828） | ＜0.001 |

HR, hazard ratio; CI, confidence interval; NLNs, negative lymph nodes.

Supplementary Table 3 Multivariate analysis of PLNs influencing CCS in patients with SBNETs

| Characteristic | | HR (95%CI) | *P* value |
| --- | --- | --- | --- |
| Race |  |  | 0.794 |
|  | White |  |  |
|  | Black | 1.024（0.753-1.391） | 0.882 |
|  | Others | 0.816（0.443-1.505） | 0.515 |
| Year of Diagnosis |  |  | ＜0.001 |
|  | 1988-1999 |  |  |
|  | 2000-2014 | 0.628（0.510-0.773） | ＜0.001 |
| Sex |  |  | 0.600 |
|  | Male |  |  |
|  | Female | 1.047（0.882-2.615） | 0.600 |
| Age |  |  | ＜0.001 |
|  | ≤60 |  |  |
|  | ＞60 | 2.183（1.829-2.605） | ＜0.001 |
| Tumor Site |  |  | ＜0.001 |
|  | Duodenum |  |  |
|  | Ileum | 0.487（0.353-0.672） | ＜0.001 |
|  | Jejunum | 0.563（0.375-0.846） | 0.006 |
| Tumor Size |  |  | 0.015 |
|  | ≤1cm |  |  |
|  | ≤2cm | 1.033（0.706-1.513） | 0.866 |
|  | ≤4cm | 1.190（0.812-1.743） | 0.372 |
|  | ＞4cm | 1.515（0.979-2.347） | 0.063 |
|  | unkonwn | 1.550（1.016-2.366） | 0.042 |
| Tumor differentiation |  |  | ＜0.001 |
|  | well differentiated |  |  |
|  | moderately differentiated | 1.800（1.284-2.524） | 0.001 |
|  | poorly differentiated | 4.244（2.718-6.628） | ＜0.001 |
|  | undifferentiated | 0.964（0.237-3.924） | 0.959 |
|  | unknown | 1.222（0.979-1.526） | 0.077 |
| T stage |  |  | ＜0.001 |
|  | T1 |  |  |
|  | T2 | 1.608（0.697-3.708） | 0.266 |
|  | T3 | 3.751（1.681-8.370） | 0.001 |
|  | T4 | 5.310（2.410-11.697） | ＜0.001 |
| M stage |  |  | ＜0.001 |
|  | M0 |  |  |
|  | M1 | 2.880（2.404-3.450） | ＜0.001 |
| PLNs |  |  | 0.452 |
|  | 0 |  |  |
|  | ＜12 | 1.124（0.885-1.427） | 0.338 |
|  | ≥12 | 0.912（0.549-1.517） | 0.724 |

HR, hazard ratio; CI, confidence interval; PLNs, positive lymph nodes.

Supplementary Table 4 Prognostic performance of different lymph node staging systems in the whole patients

| Variables | C-index (95%CI) | AIC |
| --- | --- | --- |
| PLNs(continuous) | 0.764 (0.743-0.784) | 7592.967 |
| NLNs(continuous) | 0.766 (0.745-0.786) | 7582.308 |
| RLNs(continuous) | 0.765 (0.745-0.785) | 7585.773 |
| LNR(continuous) | 0.766 (0.746-0.786) | 7578.546 |
| LODDS(continuous) | 0.765 (0.745-0.785) | 7581.853 |
| PLNs | 0.763 (0.743-0.784) | 7592.909 |
| NLNs | 0.766 (0.746-0.786) | 7575.672 |
| RLNs | 0.766 (0.747-0.787) | 7581.982 |
| LNR | 0.765 (0.745-0.785) | 7582.711 |
| LODDS | 0.766 (0.746-0.786) | 7575.154 |

CI, confidence interval;PLNs, positive lymph nodes; RLNs, resected lymph nodes; NLNs, negative lymph nodes; LNR, lymph node ratio; LODDS, the log odds of positive lymph nodes.

Supplementary Table 5 Key points of WHO classification of gastrointestinal tract neuroendocrine

| Classification/Grade | Ki-67 Proliferation Index (%) | Mitotic Index (mitoses/2 mm2) |
| --- | --- | --- |
| WHO 2010 |  |  |
| well differentiated, G1 | ＜2% | ＜2 |
| well differentiated, G2 | 3-20% | 2-20 |
| poorly differentiated, G3 | ＞20% | ＞20 |
| WHO 2019 |  |  |
| well differentiated, G1 | ＜3% | ＜2 |
| well differentiated, G2 | 3-20% | 2-20 |
| well differentiated, G3 | ＞20% | ＞20 |
| poorly differentiated, G3 | ＞20% | ＞20 |

WHO, the World Health Organization.
