# Supplementary material for: Prognostic Performance of Different Lymph Node Staging Systems in Patients With Small Bowel Neuroendocrine Tumors
Source: Front Endocrinol (Lausanne). 2020 Jul 7;11:402. doi: 10.3389/fendo.2020.00402 (PMC7358303; doi:10.3389/fendo.2020.00402)
Supplement: Supplementary file 2 [file Data_Sheet_1.ZIP › Supplementary Figure/Supplementary Figures Legends.docx]

Supplementary Fig. 1 The program selection details for the SEER database queries.

Supplementary Fig. 2 X-tile analysis was performed using patient data, equally divided into training and validation sets, from the SEER registry. The X-tile plots of training sets showed the χ2 log-rank values produced when dividing the cohort with optimal cut-point, producing high and low subsets (a). The entire cohort was divided into low risk and high risk RLN count subgroups based on the cutoff value of 11, as shown in the histogram (b). Kaplan-Meier plots were generated based on the cutoff values (χ2 = 19.9192, *P* < 0.001) (c).

Supplementary Fig. 3 X-tile analysis was performed using patient data, equally divided into training and validation sets, from the SEER registry. The X-tile plots of training sets showed the χ2 log-rank values produced when dividing the cohort with optimal cut-point, producing high and low subsets (a). The entire cohort was divided into low risk and high risk NLN count subgroups based on the cutoff value of 7, as shown in the histogram (b). Kaplan-Meier plots were generated based on the cutoff values (χ2 = 19.9192, *P* < 0.001) (c).

Supplementary Fig. 4 X-tile analysis was performed using patient data, equally divided into training and validation sets, from the SEER registry. The X-tile plots of training sets showed the χ2 log-rank values produced when dividing the cohort with optimal cut-point, producing high and low subsets (a). The entire cohort was divided into low risk and high risk LNR count subgroups based on the cutoff value of 0.4, as shown in the histogram (b). Kaplan-Meier plots were generated based on the cutoff values (χ2 = 35.9765, *P* < 0.001) (c).

Supplementary Fig. 5 X-tile analysis was performed using patient data, equally divided into training and validation sets, from the SEER registry. The X-tile plots of training sets showed the χ2 log-rank values produced when dividing the cohort with optimal cut-point, producing high and low subsets (a). The entire cohort was divided into low risk, medium and high risk LODDS count subgroups based on the cutoff value of -1.3 and -0.3, as shown in the histogram (b). Kaplan-Meier plots were generated based on the cutoff values (χ2 = 60.1332, *P* < 0.001) (c).
