# Supplementary figures and images for: Prognostic Performance of Different Lymph Node Staging Systems in Patients With Small Bowel Neuroendocrine Tumors
Source: Front Endocrinol (Lausanne). 2020 Jul 7;11:402. doi: 10.3389/fendo.2020.00402 (PMC7358303; doi:10.3389/fendo.2020.00402)

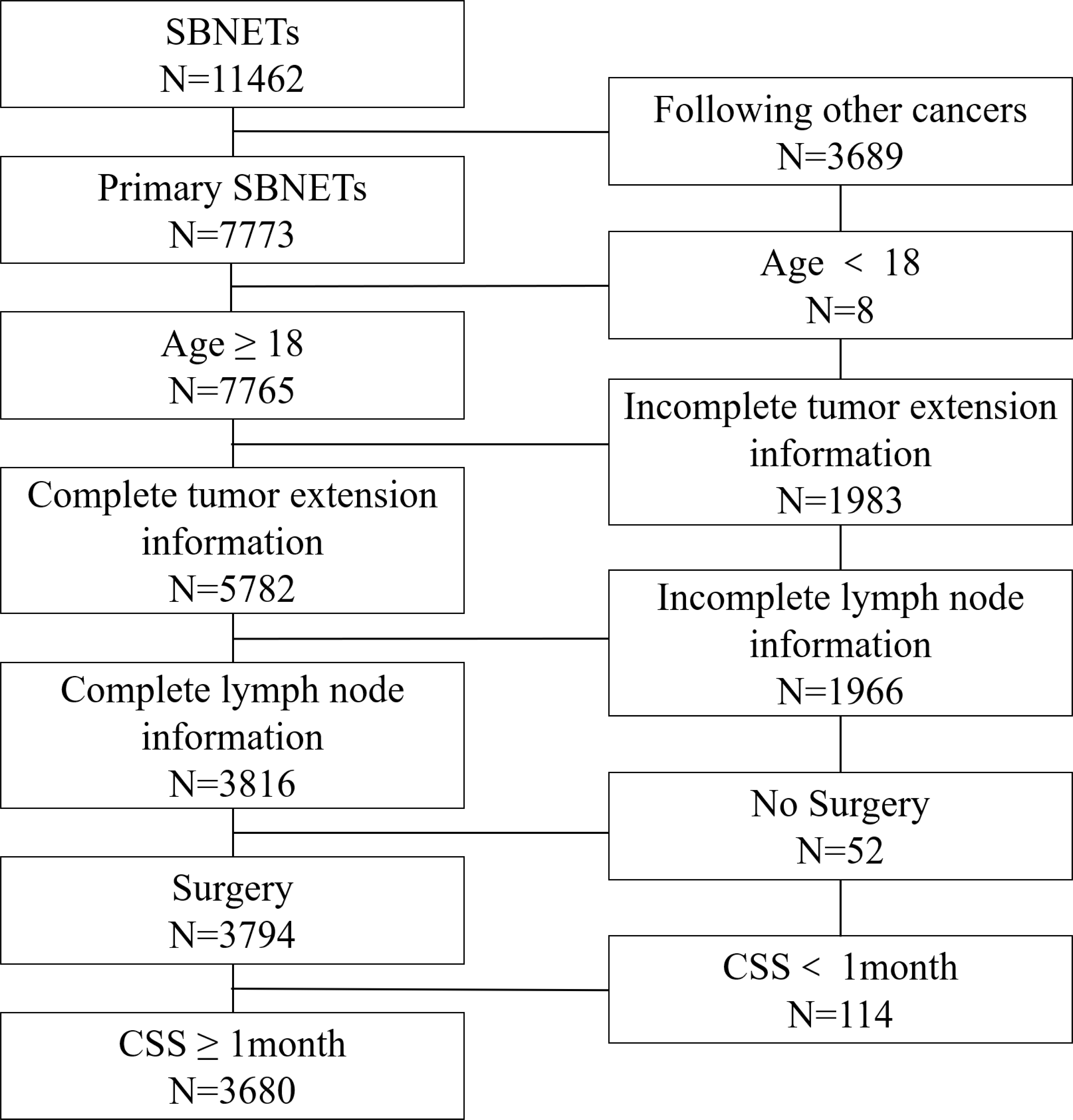

Supplement: Supplementary file 2 [file Data_Sheet_1.ZIP › Supplementary Figure/Supplementary Fig. 1.tif]

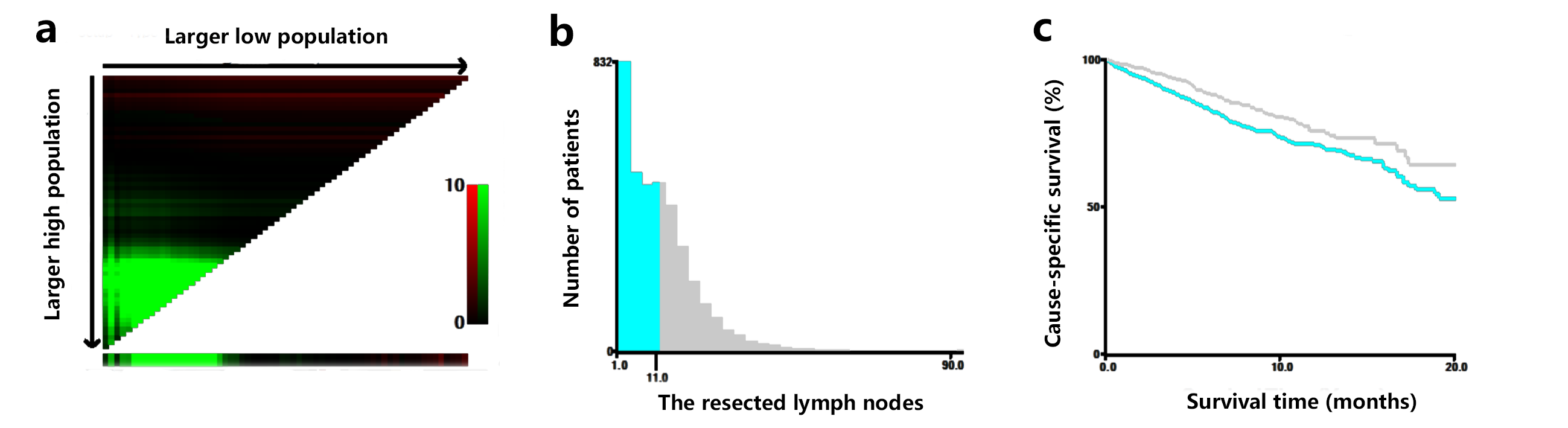

Supplement: Supplementary file 2 [file Data_Sheet_1.ZIP › Supplementary Figure/Supplementary Fig. 2.tif]

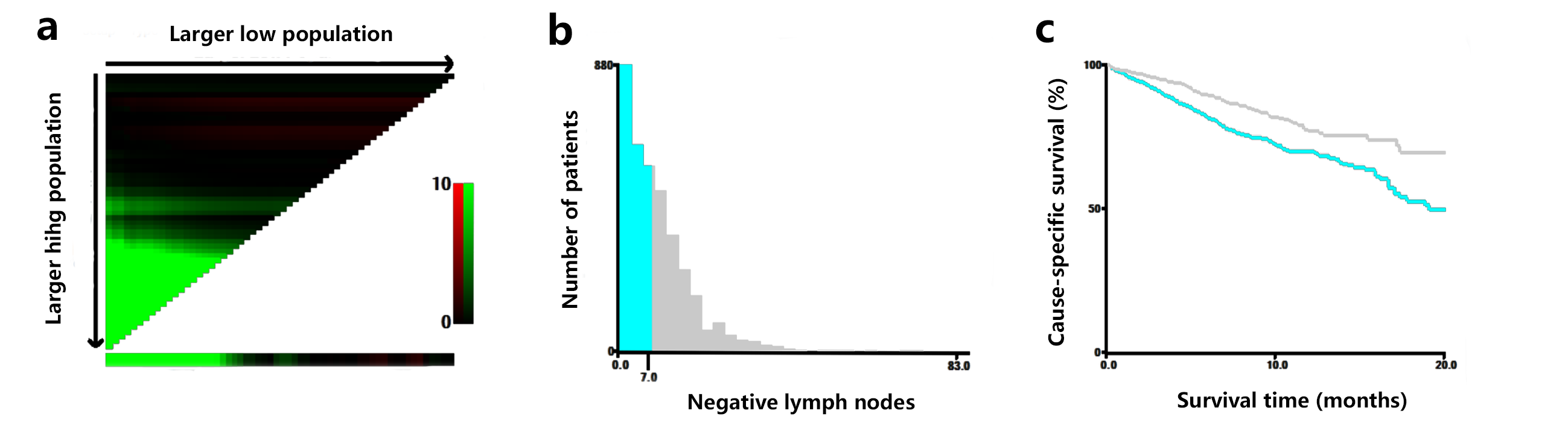

Supplement: Supplementary file 2 [file Data_Sheet_1.ZIP › Supplementary Figure/Supplementary Fig. 3.tif]

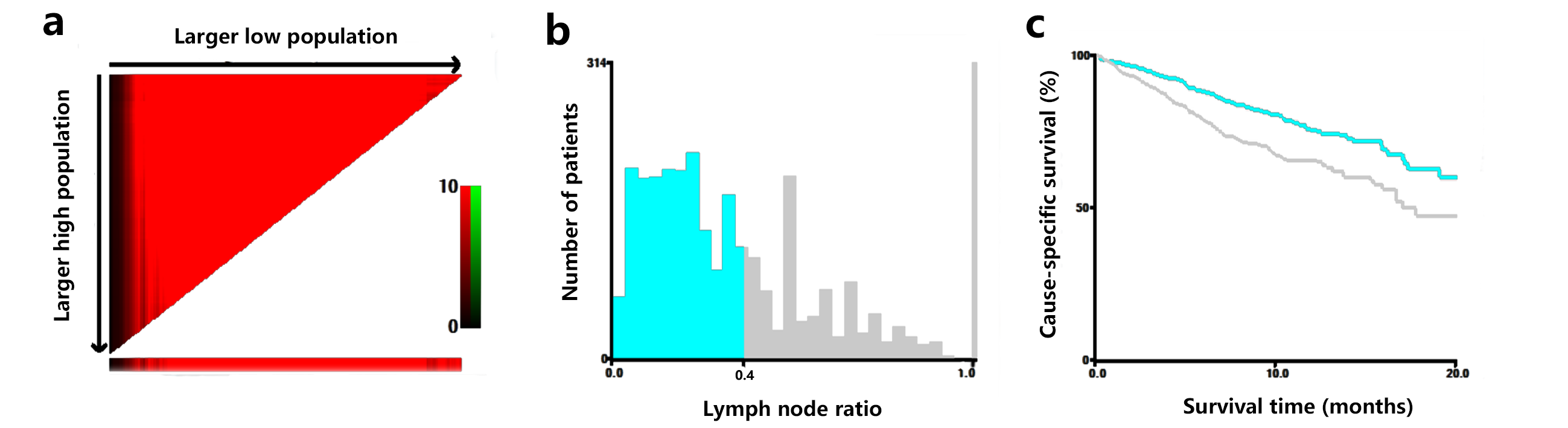

Supplement: Supplementary file 2 [file Data_Sheet_1.ZIP › Supplementary Figure/Supplementary Fig. 4.tif]

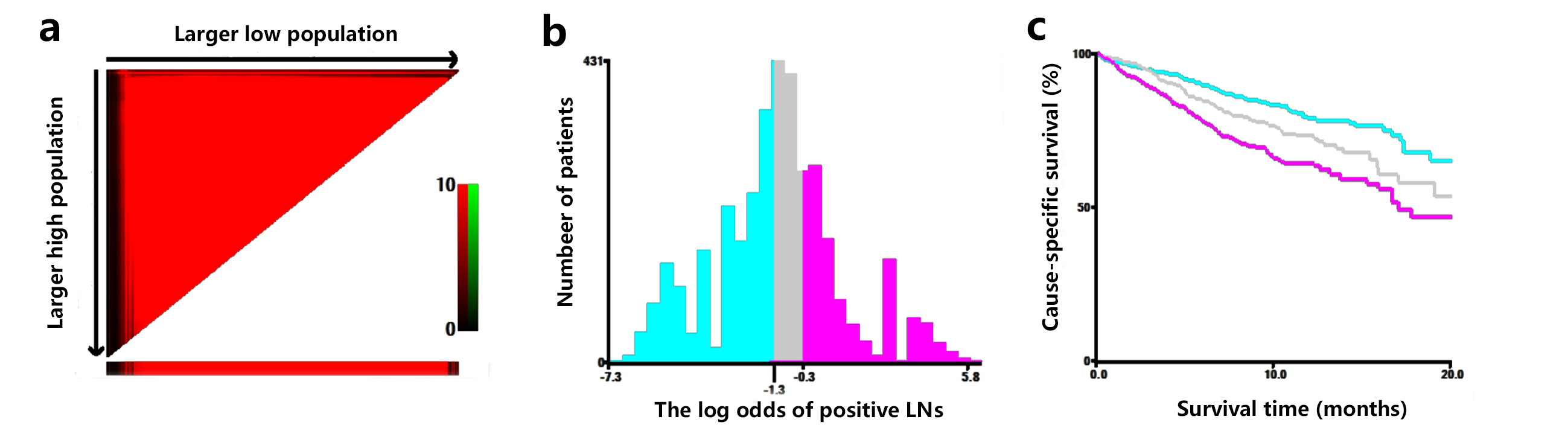

Supplement: Supplementary file 2 [file Data_Sheet_1.ZIP › Supplementary Figure/Supplementary Fig. 5.tif]
